# Identification of novel and potent dual-targeting HDAC1/SPOP inhibitors using structure-based virtual screening, molecular dynamics simulation and evaluation of *in vitro* and *in vivo* antitumor activity

**DOI:** 10.3389/fphar.2023.1208740

**Published:** 2023-07-10

**Authors:** Yingxue Yang, Shutong Chen, Qinghua Wang, Miao-Miao Niu, Yuanqian Qu, Yang Zhou

**Affiliations:** ^1^ Department of Gastroenterology, The First People’s Hospital of Kunshan, Suzhou, China; ^2^ Department of Pharmaceutical Analysis, China Pharmaceutical University, Nanjing, China; ^3^ Department of Pathology, Department of Gastrointestinal Surgery, The Affiliated Changzhou Second People’s Hospital of Nanjing Medical University, Changzhou, China

**Keywords:** cancer, histone deacetylase 1, speckle-type POZ protein, dualtargeting inhibitors, structure-based virtual screening

## Abstract

Cancer is one of the important factors threatening human health. Hence, it is essential to create novel potent drugs to treat it. Due to the strong correlation among histone deacetylase1 (HDAC1), speckle-type POZ protein (SPOP) and cancers, dual inhibition of HDAC1 and SPOP may be a promising strategy for cancer treatment. In this study, we successfully identified four potential dual-targeting HDAC1/SPOP candidate compounds with structure-based virtual screening. *In vitro* inhibition experiments confirmed that the four compounds had dual inhibitory effects on HDAC1 and SPOP. Among them, compound HS-2 had a stronger inhibitory effect on HDAC1 and SPOP than the positive controls. Further molecular dynamics simulations indicated that HS-2 could stably bind to HDAC1 and SPOP. In addition, MTT assay indicated that HS-2 inhibited the growth of tumor cells in the micromolar range. *In vivo* evaluation showed that HS-2 could obviously inhibit the growth of tumor in nude mice without obvious toxicity. These findings suggest that HS-2 is a novel and potent dual-targeting HDAC1/SPOP inhibitor for cancer treatment.

## Introduction

Cancer is a major public health problem and one of the greatest threats to human health all over the world (Siegel et al., 2022). In 2023, it is approximately estimated that 1,958,310 new cancer instances will be identified including 81,800 cases of kidney cancer and 106,970 cases of colon cancer (Siegel et al., 2023). Colon cancer has the fifth highest mortality and incidence rate in the world, accounting for one in every ten cancer cases (Han et al., 2022; Jia et al., 2022). The metastatic characteristics of both cancers are the leading cause of death in cancer patients, and patients with distant metastatic disease usually have poor survival rates (Pretzsch et al., 2019; Bray et al., 2021; Sung et al., 2021). Renal cell carcinoma (RCC) is one of the most prevalent types of malignant tumors that belong to urinary system cancer, almost representing 3% of all cancer cases (Padala et al., 2020; Han et al., 2021; Li et al., 2022). Renal cell carcinoma with clear cells (ccRCC) is the most prevalent form with a relatively poor prognosis, which accounts for more than 70% (Chen et al., 2020; Hoefflin et al., 2020; Buttner et al., 2022). Although cancer is highly resistant to chemotherapy agents, chemotherapy remains the standard of treatment for patients suffering from cancers (Che et al., 2021; Huang et al., 2022). Therefore, it seems urgent to find novel targeting agents to improve the therapeutic effect of cancers.

Histone deacetylases (HDACs) are a significant class of epigenetic enzymes that participates in cell regulation and expression of genes by taking away the acetyl groups from lysine residues of both histones and non-histones (Wang H. et al., 2020a; Lanzi et al., 2021; Zhang and Fu, 2021). To date, 18 human HDACs have been identified and classified into four groups based on their sequence homology with yeast histone deacetylase genes: class I (HDACs 1, 2, 3, and 8), class II (HDACs 4, 5, 6, 7, 9, and 10), and class IV (HDAC11) are zinc-dependent metalloproteins that hydrolyze amide bonds with water as a nucleophile, class III (Sirtuins) however, are NAD-dependent and do not respond to classical HDAC inhibitors (Liu S. S. et al., 2020b; He et al., 2020). Class I is highly expressed in tumor cells, especially HDAC1, HDAC2 and HDAC3 (Kato et al., 2009; Hoefflin et al., 2020; Que et al., 2021). HDACs cause cell cycle arrest in a p53 which acts as an oncogenic tumor suppressor protein independent manner by the transcriptional activation of cell cycle regulatory genes (Cha et al., 2009; Wang et al., 2023). Moreover, modification of p53 acetylation regulates the transcriptional activity and is associated with the control of apoptosis and autophagy, which are important for the treatment of malignant tumors (Mrakovcic et al., 2019). And HDAC inhibitors also inhibit angiogenesis and cell proliferation (Jin et al., 2021; Ou et al., 2022). Therefore, inhibition of HDACs is considered an attractive strategy for the treatment of cancers. Several HDAC inhibitors, such as Vorinostat, Panobinostat, and Romidepsin, have FDA approval for the treatment of lymphoma and myeloma (Chang et al., 2021; Lanzi et al., 2021). The clinical data on HDAC inhibitors as monotherapeutic treatments in solid tumors are poor, despite encouraging preclinical findings on these inhibitors (Pili et al., 2017; Mrakovcic et al., 2019). Therefore, we are considering the use of HDAC in combination administration for cancers.

Speckle-type POZ protein (SPOP) is a nuclear protein whose imbalance leads to abnormal regulation of cell proliferation, cell apoptosis and cell cycle (Wang Z. et al., 2020b; Clark and Burleson, 2020; Song et al., 2020). As an E3 ligase junction, SPOP can mediate E3 ubiquitin ligase recognition of substrate protein and catalyze protein ubiquitin by influencing the ubiquitin signaling pathway (Chen et al., 2016; Cho et al., 2016; Wettersten et al., 2017; Kaelin, 2022). At the same time, in the cytoplasm of ccRCC, SPOP is overexpressed, which boosts proliferation and eventually encourages tumorigenesis ultimately (Senft et al., 2018; Ju et al., 2019). In colon cancer cells, overexpression of SPOP significantly inhibits cell proliferation and migration by up-regulating E-cadherin and down-regulating Vimentin, MMP2 and MMP7 (Song et al., 2020). Most previous studies of SPOP have focused on its functions and molecular mechanisms, there have been few reports of SPOP inhibitors in cancer treatment to date (Dai et al., 2017; Zhang et al., 2017; Zhang et al., 2021). A small molecule SPOP inhibitor, SPOP-IN-6b has been reported to inhibit both the activity of SPOP and the carcinogenic signaling pathways and finally kill human ccRCC cells which depend on carcinogenic cytoplasmic SPOP (Guo et al., 2016). However, SPOP-IN-6b showed inhibitory activity in the micromolar range in both *in vivo* and *in vitro*, and its affinity and activity were low. Meanwhile, the clinical therapeutic efficacy remained to be explored (Guo et al., 2016). As a result, there is an urgent need to develop potent small molecule inhibitors of SPOP.

Although HDAC inhibitors have manageable side effects and are well tolerated by patients, they do not have enough activity for cancer therapy (Ho et al., 2020; Rausch et al., 2020). The combination of HDAC inhibitors with other anticancer agents is of great benefit in the treatment of cancers in a growing number of studies (Kiweler et al., 2020; Molina et al., 2020; Pili et al., 2020). They can enhance the inhibition of cell proliferation and cell growth, and also enhance the inhibition of angiogenesis in cancers (Rausch et al., 2020; Sharma et al., 2021). However, combination therapies may cause undesirable drug interactions and unnecessary drug toxicity (Zou et al., 2019; Ince and Eisen, 2021). To avoid the mentioned problem, we intend to combine HDAC1 with other targets to develop dual-targeting inhibitors. Even though SPOP serves as a regulatory hub in the treatment of cancers, few SPOP inhibitors are reported to date. Therefore, this study aims to develop novel potent HDAC1/SPOP dual-targeting inhibitors to avoid the limitations of single-target therapy and improve therapeutic efficacy.

Structure-based virtual screening is a useful and valuable silico technique and is generally recognized as an important method for finding new drug compounds (Zhou et al., 2021b; Sanachai et al., 2022). Compared with traditional screening methods, structure-based virtual screening improves the efficiency of screening large-scale compound databases and also provides more reasonable and accurate binding modes (Zhou et al., 2021a; Sirous et al., 2021). In previous studies, kinds of research on single-targeting inhibitors of HDAC or SPOP using structure-based virtual screening has been reported (Guo et al., 2016; Liu J. et al., 2020a; Sirous et al., 2020). Although virtual screening techniques have been utilized to identify SPOP or HDAC1 single-targeting inhibitors, there are currently no reports of dual-targeting HDAC1/SPOP inhibitors (Guo et al., 2016; Liu J. et al., 2020a). In this study, taking full advantage of the aforementioned virtual screening, we successfully identified the first dual-targeting HDAC1/SPOP inhibitor.

Throughout the study, we first preprocessed the ligands obtained from the Protein Data Bank were preprocessed. Secondly, PAINS filtering and drug-likeness screening were used to get the hit compounds, which were docked to HDAC1 and SPOP separately to screen potential dual-targeting compounds. Finally, four compounds with good docking scores were selected for the inhibitory activity assay, and the compounds with the best activity were selected for further biological evaluation to determine their anti-tumor activity.

## Materials and methods

### Materials

All tumor cell lines including HCT-116, A498, 769-P, Caki-2, Ramos, OS-RC-2 and Ketr-3 cells were obtained from the American Type Culture Collection (ATCC, Manassas, VA, United States). All cells were maintained in a humidified incubator with 5% CO_2_ and 95% air at 37 °C under standard conditions. Compounds were purchased from WuXi AppTec. HDAC1 and SPOP proteins were obtained from Abcam (Cambridge, MA, United States).

### Docking-based virtual screening

Docking-based virtual screening was performed using the molecular docking module in Molecular Operating Environment (MOE) Program. The X-ray 3D structures of HDAC1 (PDB ID: 5ICN) and SPOP (PDB ID: 3HQL) were obtained from the Protein Data Bank. In front of docking, the two proteins were prepared with the QuickPrep function in MOE program to optimize their protonation states and add hydrogen atoms. An in-house database contains 43,000 compounds. All these compounds are composed of 8,000 compounds from the commercially available SPECS database (http://www.specs.net) and 35,000 compounds reported in previous studies (Zhou et al., 2019). A total of 43,000 compounds from an in-house database were further converted into three-dimensional structures through the energy optimization algorithm of MOE and used for virtual screening (Zhou et al., 2019). The PAINS-Remover program was used to screen and remove Pan Assay Interference Compounds from the virtual screening compound database and remove these compounds in biological analysis to reduce the false positive rate of the compounds (Baell and Holloway, 2010). A total of 42,510 compounds were obtained after filtration. According to the modified drug-likeness properties, which means the molecular weight ≤600, the number of hydrogen bond donors ≤5, the number of hydrogen bond acceptors ≤10, the log P (log octanol/water partition coefficient) ≤5, SwissADME was used to filter 37,060 compounds for further virtual screening (Daina et al., 2014; 2017). After that, the docking validation was performed to demonstrate the similar congruence between the native and the docked pose. All compounds were connected to the HDAC1 active site by the Dock protocol of the MOE program and potential HDAC1-targeting compounds were screened by a reasonable Docking Scoring threshold, resulting in 117 compounds with binding free energy <−14.05 kcal/mol. Finally, the selected compounds were paired to the active site of SPOP, and then the optimal four compounds with potential dual-targeting HDAC1/SPOP were selected for further biological evaluation.

## Enzymatic assays for HDAC1 and SPOP

*In vitro* HDAC1 inhibition experiments were carried out as described previously (Li et al., 2014). In the 96-well plates, different concentrations of the tested compound (50 μL) were added to 10 μl of HDAC1 enzyme solution. After incubation at 37°C for 5 min, 40 μl of the fluorescent substrate Boc-Lys (acetyl)-AMC was then added to the mixture. The plates were incubated at 37°C for 0.5 h, and the developer (100 μl) containing trypsin and TSA was added to the mixture. Finally, the fluorescence intensity at wavelengths of 390 nm and 460 nm was measured 20 min later by a microplate reader (Perkin Elemer).

The enzymatic activity of SPOP was determined by a previously described fluorescence polarization (FP) assay (Guo et al., 2016). FITC-labeled peptide substrate puc_SBC1 (FITC-puc_SBC1, FITC-LACDEVTSTTSSSTA) was purchased (GL Biochem Ltd, Shanghai). Serial dilutions of competitors for SPOP23-337 were prepared from the 20 mM DMSO stocks. The diluted compound was added to the reaction mixture in 100 μl of HEPES (25 mM, pH 7.5) containing 10 μM SPOP and 50 nM FITC-puc_SBC1. After incubation for 1 h at 4°C, FP was measured on a microplate reader (Perkin Elemer) using the wavelengths of 480 nm for excitation and 535 nm for emission, respectively.

All experiments were performed in triplicate. Competition binding data were analyzed using GraphPad Prism 6.0 software (GraphPad Software Inc, San Diego, CA) and the inhibition constants (IC_50_) were calculated by nonlinear curve fitting.

### Molecular dynamics simulation

The GROMACS (version 2021.5) program with periodic boundary conditions was used to simulate the MD of HS-2 in the AMBER99SB-ILDN force field to analyze the changes in system stability over time (Abraham et al., 2015; Pall et al., 2020). Firstly, single point charge (SPC) water molecules were used to solvate the compound in a cube box with 1.0 nm away from the compound, and Na^+^ and Cl^−^ were used to replace the water molecules to neutralize the energy of the system. It then used the steepest descent algorithm and set 5,000 steps to minimize the energy system. A further NVT simulation of 100 ps was performed with a V-rescale thermostat to keep the system temperature at 300 K. The Parinello-Rahman regulator was then simulated with 100ps NPT to maintain the system pressure of 1 bar. Finally, the system was conducted to 50 ns MD simulation and the root mean square deviation (RMSD) and root mean square fluctuation (RMSF) analysis were recorded. 10 ps time intervals were used to save the trajectory data.

### Cell growth inhibitory activity

According to the previously reported method (Namwan et al., 2022; Zhao et al., 2023), at a concentration of 5 × 10^4^/ml, cell lines were planted in 96-well plates and cultivated for an entire night. The cells were incubated at 37 °C for 72 h while being exposed to various inhibitor concentrations. And then, MTT stock solution (5 mg/ml) was added to each well and incubated for an additional 4 h. Dimethyl sulfoxide (DMSO) was used to dissolve the insoluble crystals, and a microplate reader was used to detect absorbance at 570 nm. Survival ratio (%) was calculated using the following equation: survival ratio (%) = (*A*_*treatment*_/*A*_*control*_) × 100%. Data were analyzed using the analysis software GraphPad Prism 6.0 software (GraphPad Software Inc., San Diego, CA), and the half-maximal inhibitory concentration (IC_50_) values were calculated by the dose-response curve visualized using nonlinear regression (curve fit).

### Western blot analysis

The tumor cells (10^6^ cells/well) were seeded onto 6-well plates and allowed to culture for 24 h. The cells were incubated with various concentrations (0, 0.5 and 2 μM) of HS-2 at 37°C for 72 h. Tumor cells cells were washed and lysed with RIPA. Protein samples were detected by western blot as described previously (Zheng et al., 2021).

### *In vivo* anticancer activity

According to the previously reported *in vivo* inhibition method, we injected HCT-116 tumor cells (200 μl, 1 × 10^7^ cells) into the right subcutaneous space of 6-week-old BALB/c nude mice (Experimental Animal Center of Yangzhou University (Yangzhou, China) (Zheng et al., 2021). All experimental protocols were approved by the Animal Ethics Committee of China Pharmaceutical University. When the tumors grew to 90–120 mm^3^, the mice were randomly divided into three groups (five mice per group) and intraperitoneally administered with vehicle (PBS buffer pH 7.4, volume capacity: 0.2 ml), compound HS-2 (volume capacity: 0.2 ml in PBS vehicle, 5 mg/kg), and compound HS-2 (volume capacity: 0.2 ml in PBS vehicle, 10 mg/kg) every 3 days for a total of six times. Tumor volume and weight were measured every 3 days. Tumor volume was calculated using the formula (c × c × d)/2 (c, the smallest diameter; d, the largest diameter).

## Results

### Docking-based virtual screening

**FIGURE 1:**
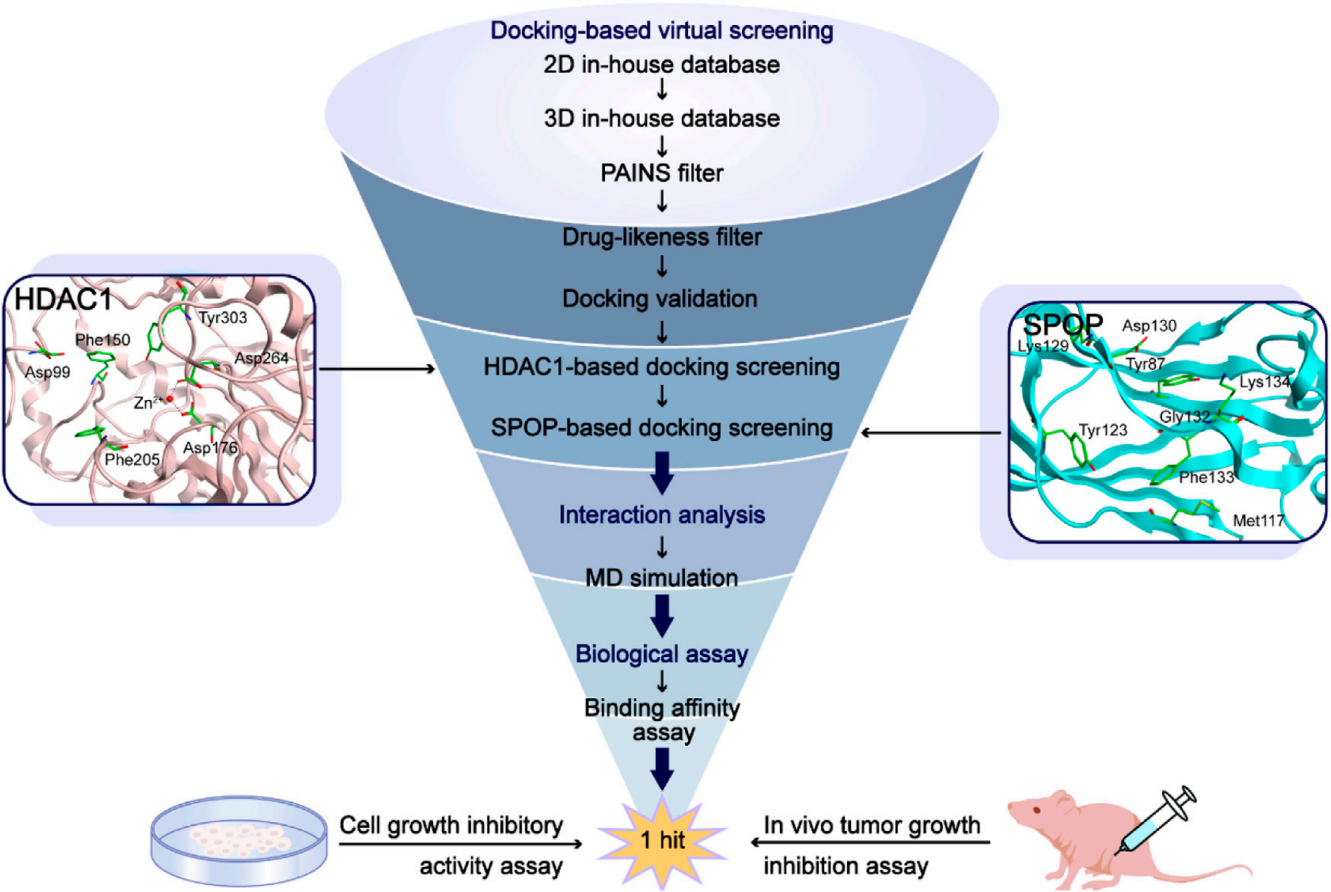
The workflow of the docking-based virtual screening process of dual-targeting HDAC1/SPOP inhibitors.

**FIGURE 2:**
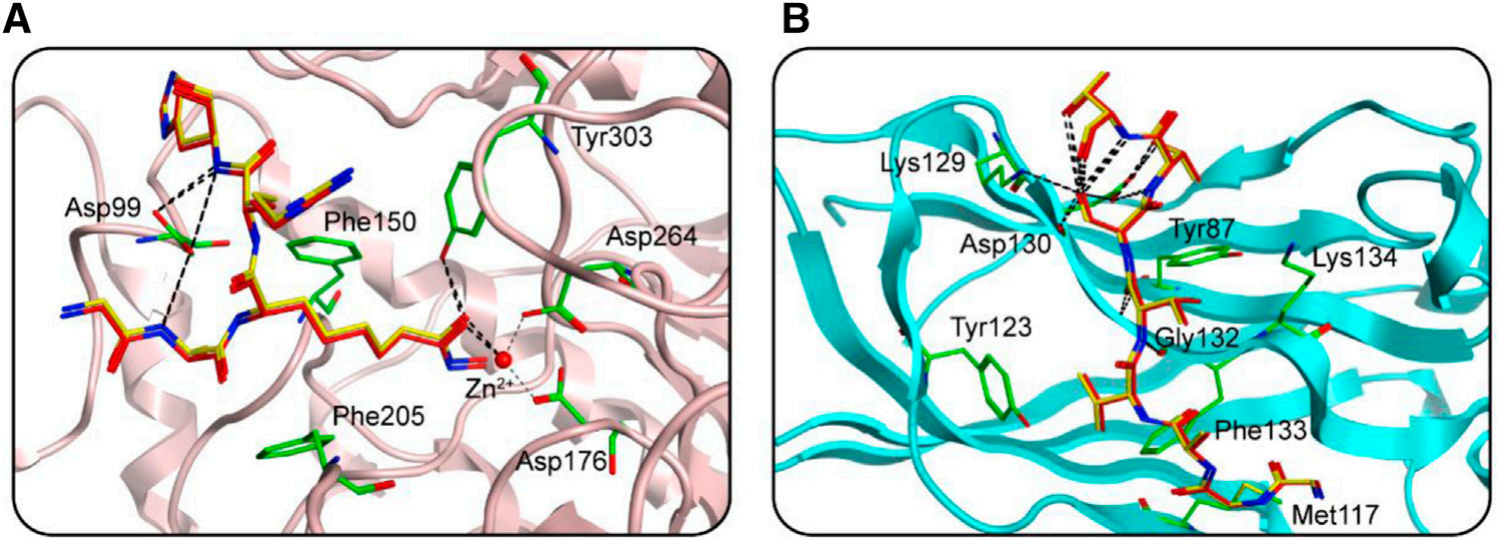
The docking validation before molecular docking. **(A)** The docking validation of HDAC1. **(B)** The docking validation of SPOP. (yellow for docking pose, red for native pose).

**FIGURE 3:**
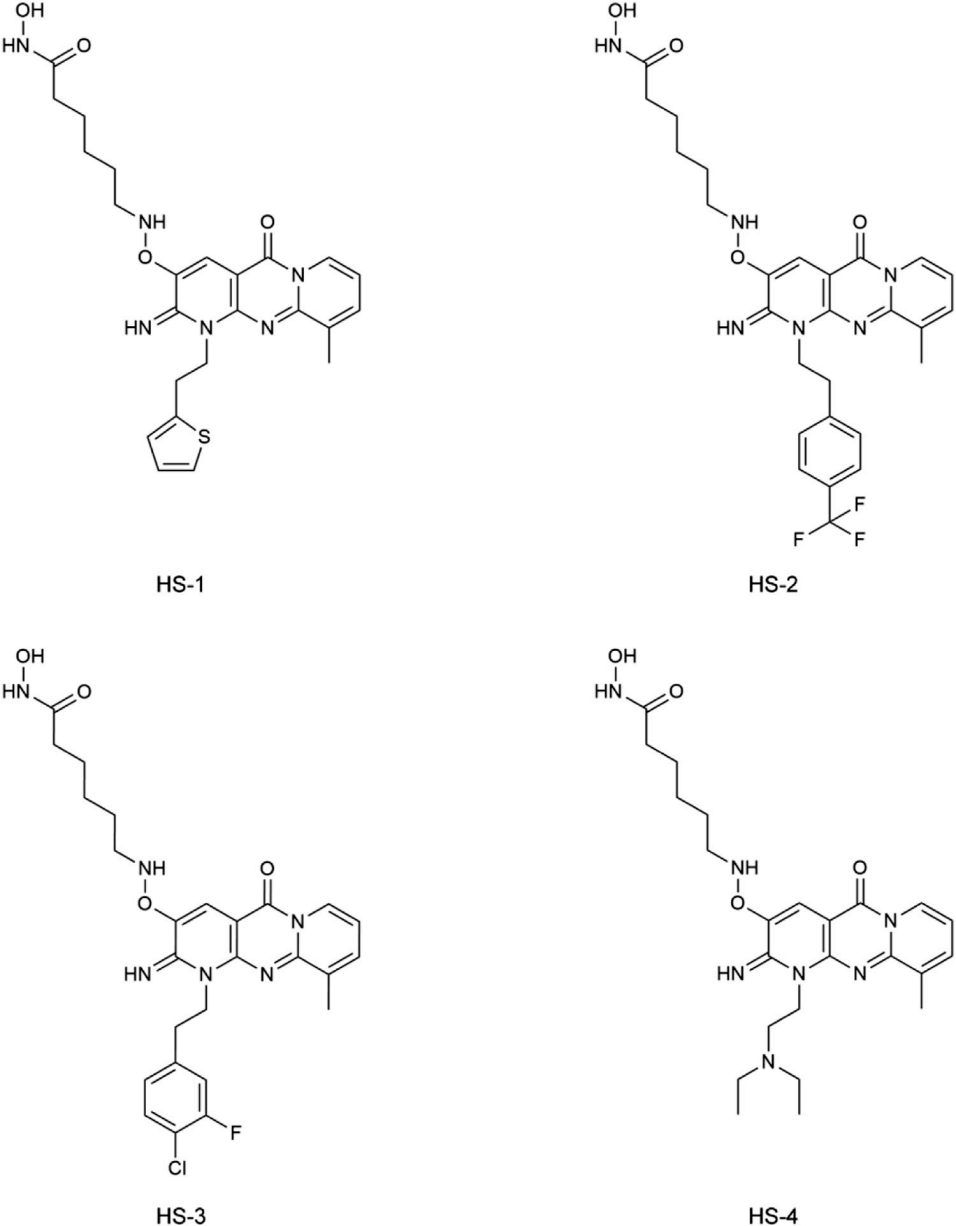
The chemical structures of four compounds.

In this study, potential dual-targeting HDAC1/SPOP inhibitors were identified from an in-house database, and the multi-step process of docking-based virtual screening was shown in Figure 1. A total of 43,000 compounds were processed for Energy Minimization in MOE, and the 2D structure of each compound was transformed into the 3D structure to prepare the 3D database. After that, the PAINS filter was applied to reduce the false positive rate of compounds and improve the efficiency of virtual screening. According to the modified drug-likeness properties as the basis for filtering to narrow down the filtering scope. Before docking, the validation of docking was performed in HDAC1 and SPOP respectively. The validation results were shown in Figures 2A, B, indicating the native and docking poses of both HDAC1 and SPOP are almost identical. Then, the Dock program was used to perform docking-based virtual screening of HDAC1 (PDB ID: 5ICN) and SPOP (PDB ID: 3HQL). The binding affinity between each compound and a target was determined by the size of the binding free energy. In general, a lower binding free energy indicated a stronger binding affinity. The peptide inhibitor of the HDAC1 crystal structure (PDB ID: 5ICN) was chosen as the positive control with a docking score of −14.05 kcal/mol. According to the scoring threshold of −14.05 kcal/mol, 117 compounds were obtained. Afterward, the 117 compounds below −14.05 kcal/mol were further docked into the active site of SPOP, and SPOP-IN-6b was selected as the positive control with the docking score of −8.40 kcal/mol. According to the scoring threshold of −8.40 kcal/mol, four potential dual-targeting candidate compounds simultaneously satisfying both scoring thresholds were obtained (Table 1). The structures of the compounds were shown in Figure 3. Finally, the drug-likeness parameters of 4 compounds were calculated (Table 2) and further biological research was carried out.

**TABLE 1:** The docking scores and biological data of four hit compounds.

Name	HDAC1	SPOP	HDAC1 (IC_50_, nM)	SPOP (IC_50_, μM)
	Binding free energy^a^ (kcal/mol)	Binding free energy (kcal/mol)		
HS-1	−14.15	−8.51	13.7 ± 1.1	18.3 ± 2.5
HS-2	−14.34	−8.94	7.6 ± 0.4	9.1 ± 0.7
HS-3	−14.21	−8.76	11.5 ± 1.3	14.9 ± 1.2
HS-4	−14.09	−8.43	18.4 ± 1.9	23.2 ± 2.7
SPOP-IN-6b	-	−8.40	no binding	34.6 ± 3.1
Vorinostat	-	-	8.3 ± 1.4	no binding

^a^ Binding free energy between the compound and the target (lower binding free energies show stronger binding affinities).

**TABLE 2:** The drug-like parameters of four compounds.

Name	Molecular weight	nHA	nHD	logP
HS-1	496.58	7	4	3.84
HS-2	558.55	10	4	4.21
HS-3	542.99	8	4	3.48
HS-4	485.58	8	4	3.85

### Interaction analysis

**FIGURE 4:**
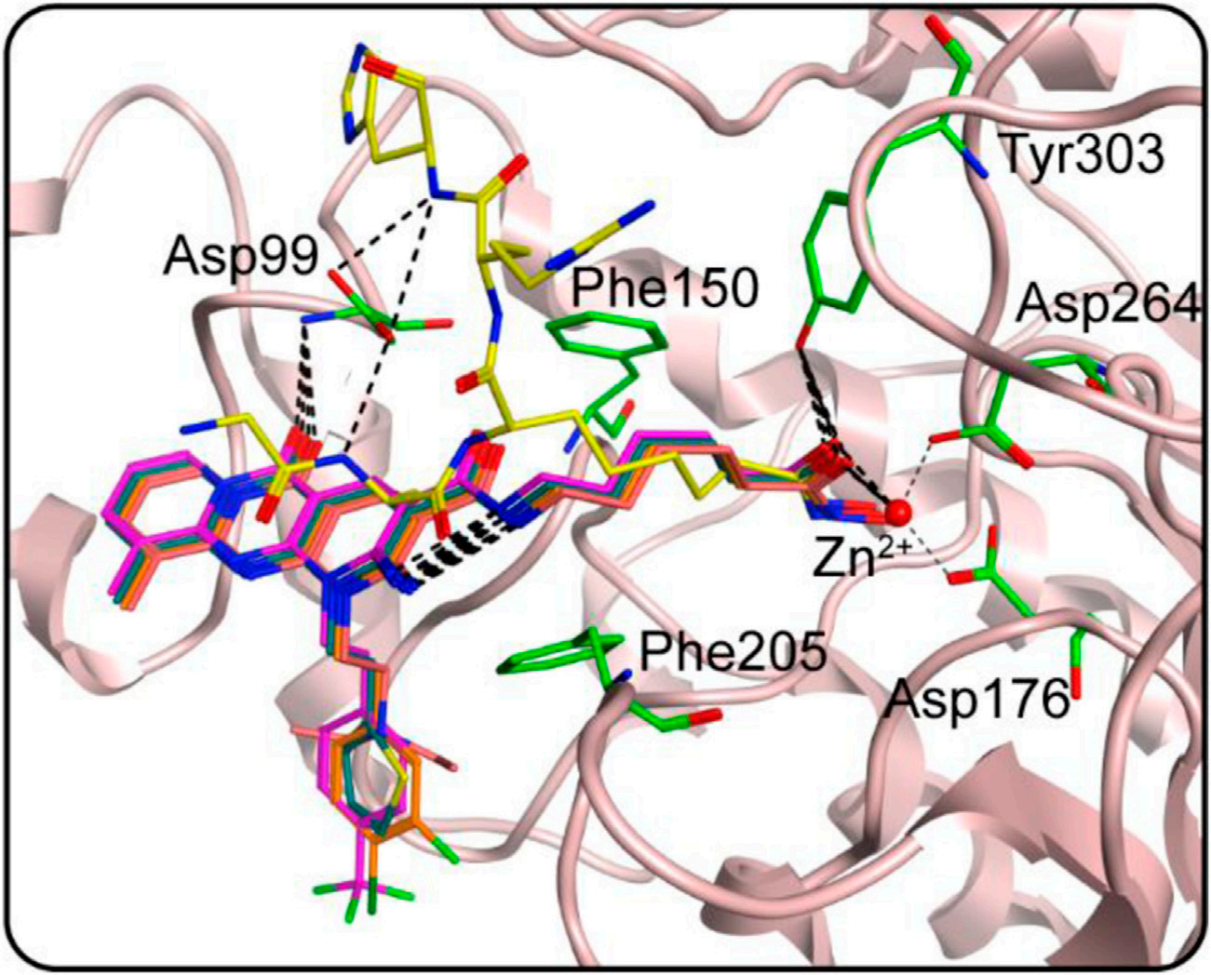
The docking poses of HS 1–4 at the HDAC1 active site (yellow for native ligand, dark cyan for HS-1, magenta for HS-2, orange for HS-3 and dark pink for HS-4). Hydrogen bonds are indicated by dashed black lines.

**FIGURE 5:**
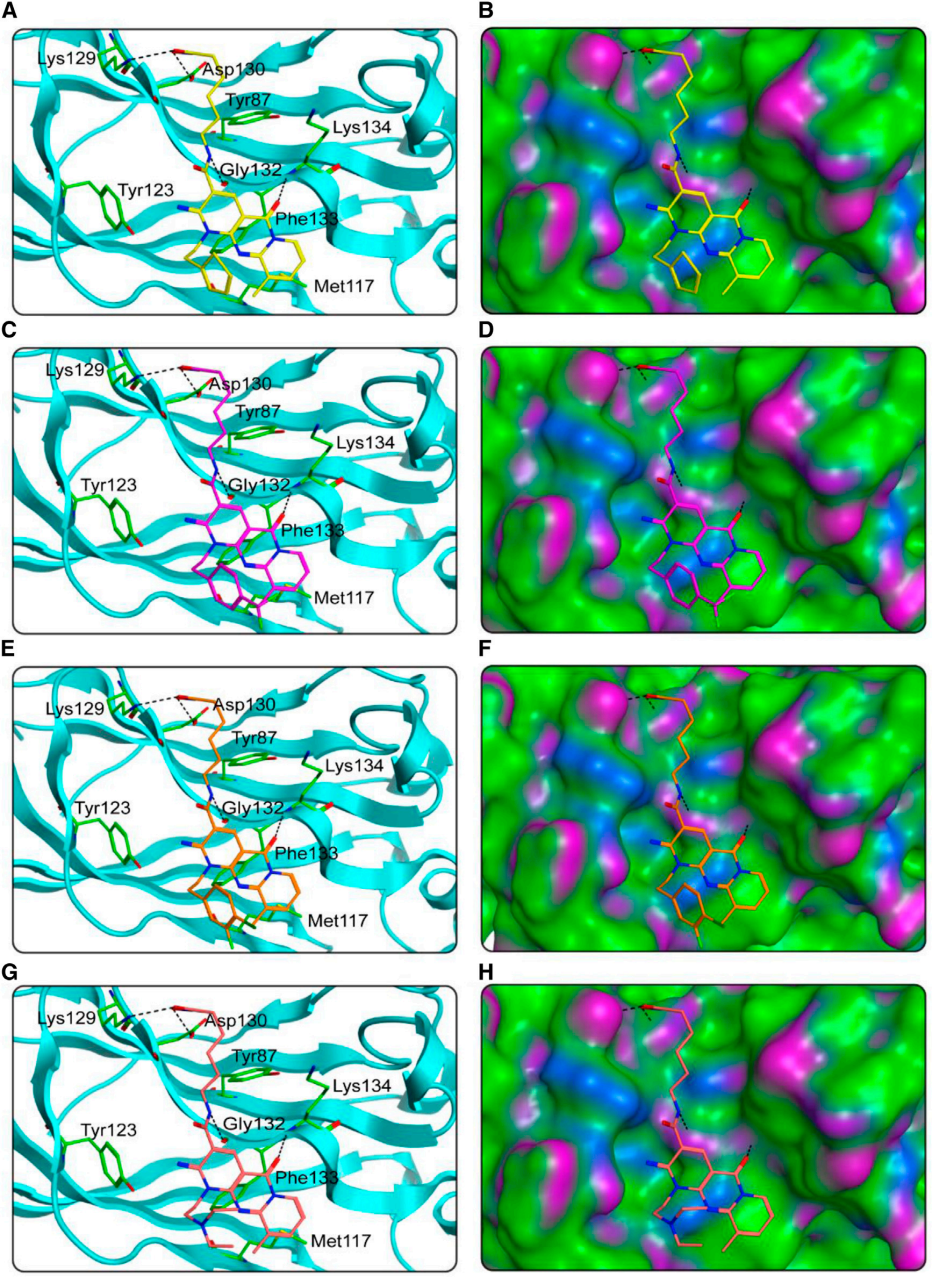
The docking poses of HS 1–4 at the SPOP active site were predicted. **(A)**, **(B)** HS-1 and its corresponding surface map; **(C, D)** HS-2 and its corresponding surface map; **(E, F)** HS-3 and its corresponding surface map; **(G, H)** HS-4 and its corresponding surface map. Compounds are indicated by different colors (yellow for HS-1, purple for HS-2, orange for HS-3, and pink for HS-4), and SPOP is coded by cyan-blue. Hydrogen bonds are indicated by dashed black lines. The surface of SPOP is plotted by H-bond (purple), hydrophobic (green), and mildly polar (blue) regions.

We studied the binding mode of HS 1–4 to the active sites of HDAC1 and SPOP. Figure 4 showed the docking and binding mode between HS 1–4 and HDAC1. In Figure 4, the compounds HS 1–4 were strongly coincident, and six amino acid residues (Asp99, Phe150, Asp176, Phe205, Asp264, and Tyr303) played an important role in the specific binding of each compound to HDAC1. As with the interaction between the native ligand and the zinc ion, the end-hydroxamic acid structure of each compound formed ionic bonds with the zinc ion, which also did the same with Asp176 and Asp264. Additionally, the compounds could establish hydrogen bonds with Asp99 and Tyr303, respectively. Ionic and hydrogen bonds could stabilize the binding of the compound to the active site and help to enhance the reactivity of the compound. It could be concluded that the screened compounds could be well combined with HDAC1. Figure 5 exhibited the docking and binding mode of HS 1–4 and SPOP. As shown in Figures 5A, C, E, G, HS 1–4 formed hydrogen bonds with eight amino acid residues, namely Tyr87, Met117, Tyr123, Lys129, Asp130, Gly132, Phe133 and Lys134, respectively. In addition, it could be seen from Figures 5B, D, F, H that the compound could be perfectly accommodated by the active pocket of SPOP. In summary, the docking results of HS 1–4 with HDAC1 and SPOP fully indicate that HS 1–4 interact with key amino acid residues of both HDAC1 and SPOP, thus demonstrating that HS 1–4 might be a potential dual-targeting inhibitor.

### Inhibition of HDAC1 and SPOP by HS 1–4

To further assess the inhibitory effects of four selected compounds on the dual targets in further detail, *in vitro* inhibition experiments of four selected compounds were performed. SPOP-IN-6b and Vorinostat were selected as positive controls, respectively. As shown in Table 1, HS 1–4 showed inhibitory effects on both targets. The IC_50_ values of HDAC1 inhibition activity of HS 1–4 were 13.7, 7.6, 11.5, and 18.4 nM, and the IC_50_ values of SPOP inhibition activity of HS 1–4 were 18.3, 9.1, 14.9, and 23.2 μM, respectively. In the positive control, SPOP-IN-6b showed an inhibitory activity on SPOP without an inhibitory effect on HDAC1, while Vorinostat showed an inhibitory activity on HDAC1 without affecting SPOP activity. It was worth noting that among the four compounds, compound HS-2 had the strongest inhibitory activity against HDAC1 and SPOP. The inhibitory effect of HS-2 was stronger than that of the positive controls, and its IC_50_ value is about 1.1 times that of Vorinostat and 3.8 times that of SPOP-IN-6b. Therefore, the most promising inhibitor HS-2 was further used for molecular dynamics simulation.

### Molecular dynamics simulation

**FIGURE 6:**
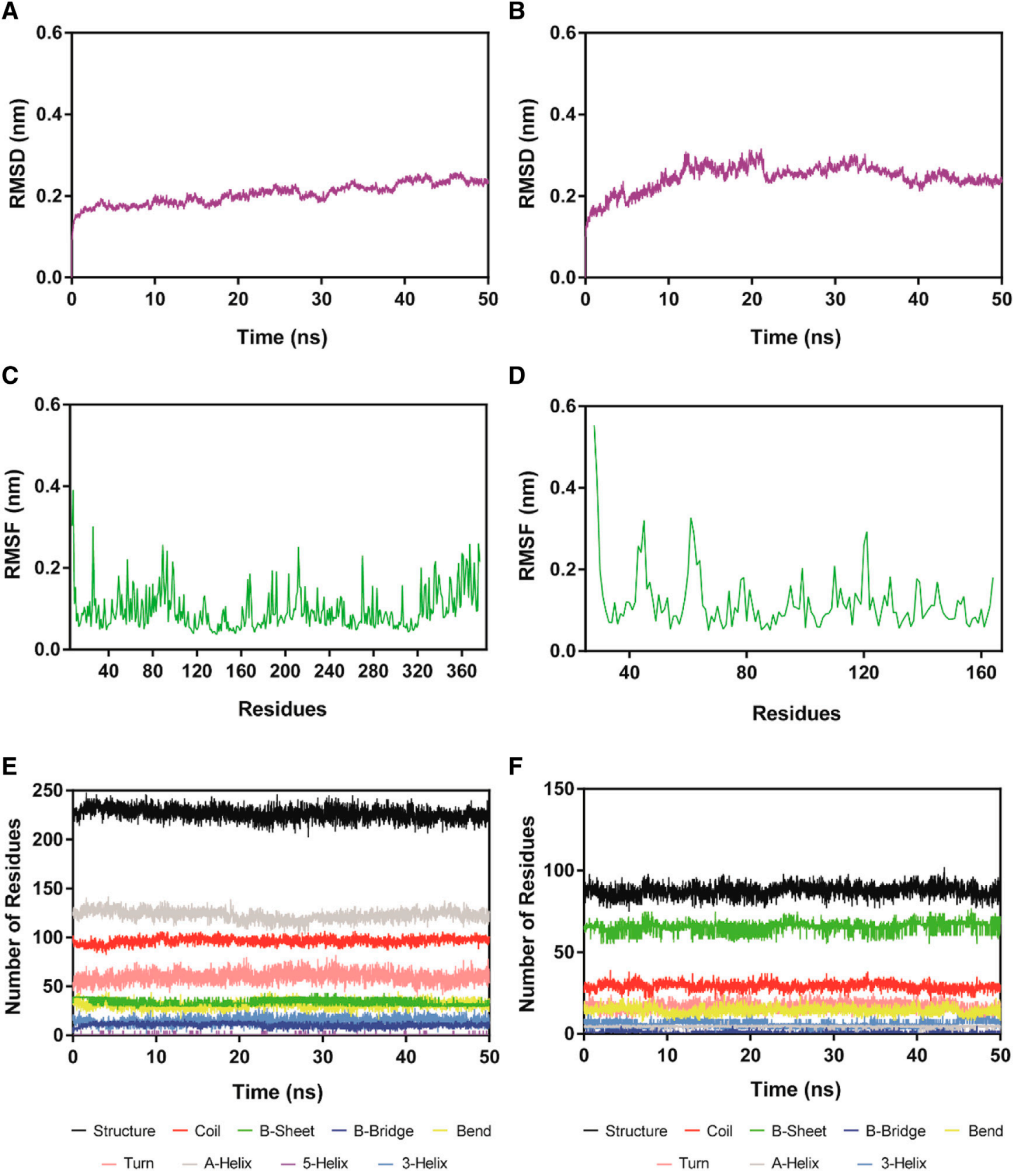
RMSD and RMSF of Cα atoms of HS-2-HDAC1 and HS-2-SPOP complexes and the secondary structures analysis of the complexes. **(A, B)** are the RMSD of HS-2 in complex with HDAC1 and SPOP, respectively. **(C, D)** are the RMSF of HS-2 in complexes with HDAC1 and SPOP, respectively. **(E, F)** are the secondary structures analysis of HS-2 in complex with HDAC1 and SPOP, respectively (black for Structure, red for Coil, green for B-Sheet, dark blue for B-Bridge, yellow for Bend, pink for Turn, grey for A-Helix, purple for 5-Helix and blue for 3-Helix).

HS-2-HDAC1 complex and HS-2-SPOP complex were simulated with 100 ns molecular dynamics to analyze the system binding stability with time. Figures 6A, B described the root mean square deviation (RMSD) of HS-2 in HDAC1 and SPOP complexes, respectively. RMSD is one of the most important factors to describe the stability of the MD simulation system. A lower RMSD value usually indicates better binding stability. The final equilibrium values of the HS-2-HDAC1 and HS-2-SPOP complexes were both below 0.4 nm, with mean RMSD values of roughly 0.20 and 0.26 nm, correspondingly. This demonstrates that HS-2-HDAC1 and HS-2-SPOP complexes are stable in molecular dynamics simulations and that HS-2 may bind to HDAC1 and SPOP concurrently and firmly. The RMSF of the amino acid residue C atom is then determined, and the stability of the system was analyzed by the flexibility of the amino acid residue. The RMSF value is typically associated with interactions between HDAC1/SPOP and HS-2, such as hydrogen bonding. In general, a low RMSF indicates minimal residue movement and consequently a stable system. As shown in Figures 6C, D, the high fluctuations of RMSF of the two complexes existed at the C-/N- ends which might be related to their few interactions. Furthermore, the key amino acid residues corresponding to HS-2-HDAC1 and HS-2-SPOP complexes showed limited fluctuations below 0.2 nm. The secondary structures of the complexes were represented in Figures 6E, F as Coil, B-Sheet, B-Bridge, Bend, Turn, A-Helix, 3-Helix, and 5-Helix in HS-2-HDAC1. These secondary structures were in a stable state of fluctuation within 50 ns. In conclusion, the results of the molecular dynamics simulation suggest that HS-2 may be firmly attached to the active pockets of HDAC1 and SPOP. Therefore, HS-2 could be a novel and effective dual-targeting inhibitor of HDAC1/SPOP.

### Cell growth inhibitory activity

To analyze the inhibitory effect of compound HS-2 on the growth of cancer-related cells furtherly, the IC_50_ value of HS-2 was determined by MTT assay. In general, a lower IC_50_ value indicated a stronger apoptosis-inducing ability of the drug. As shown in Table 3, HS-2 exhibited growth inhibitory activity on the Ketr-3 cell, A498 cell, 769-P cell, Caki-2 cell, Ramos cell, OS-RC-2 cell, and HCT-116 cell with IC_50_ values < 10 μM. Among them, HS-2 showed excellent anti-tumor activity against A498 cell, Caki-2 cell, and Ramos cell with IC_50_ values of 1.6, 1.8, and 1.9 μM, respectively. The results indicate the presence of the anti-tumor ability of HS-2. The SPOP and HDAC levels were further measured through western blot. We found that the SPOP and HDAC levels in HCT-116 and A498 cells was reduced upon the treatment of HS-2 (Supplementary Figures S1, S2).

**TABLE 3:** Cell growth inhibition of HS-2.

Assay	IC_50_ (μM)
HCT-116 cell proliferation	2.7 ± 0.8
A498 cell proliferation	1.6 ± 0.3
769-P cell proliferation	5.2 ± 1.1
Caki-2 cell proliferation	1.8 ± 0.4
Ramos Cell proliferation	1.9 ± 0.5
OS-RC-2 cell proliferation	9.3 ± 1.7
Ketr-3 cell proliferation	4.1 ± 0.6

### *In vivo* tumor growth inhibition

**FIGURE 7:**
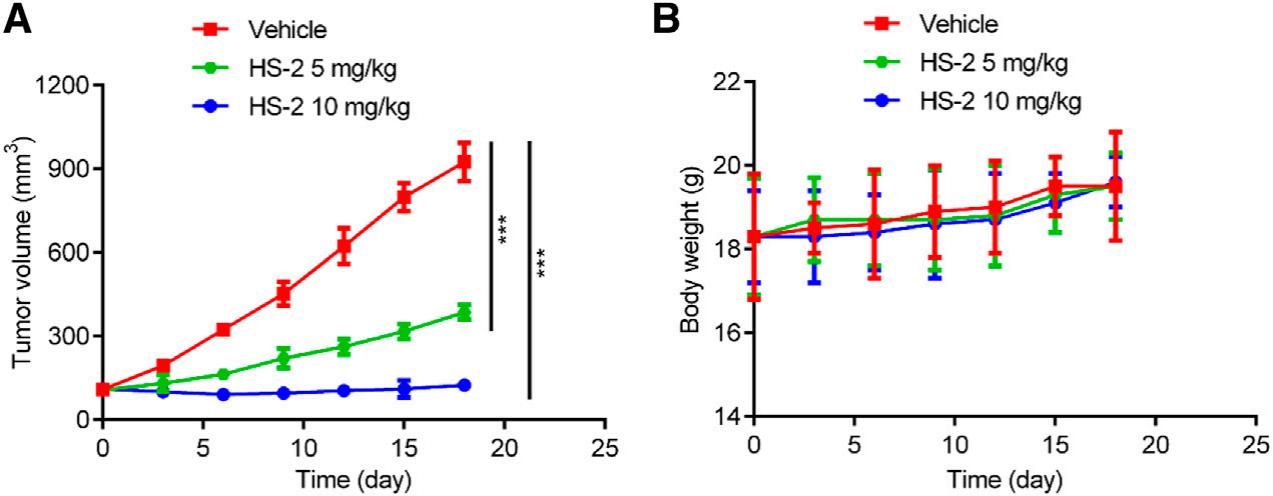
HS-2 shows strong antitumor activity to tumor cell-derived xenograft. **(A)** Changes in tumor volume. **(B)** Body weight of mice. The data are shown as mean ± SD, n = 5. ***p < 0.001 means a significant difference *versus* the vehicle group.

To further demonstrate the anti-tumor ability of HS-2 *in vivo*, HCT-116 cells were transplanted into BALB/c nude mice to establish colon carcinoma models, and the model mice were divided into three groups: vehicle, compound HS-2 (5 mg/kg), and compound HS-2 (10 mg/kg). According to Figure 7A, mice given HS-2 at doses of 5 mg/kg and 10 mg/kg had significantly less tumor volume than the control group, indicating that HS-2 exerted a significant anti-tumor effect *in vivo*. In addition, mice treated with HS-2 at the dose of 10 mg/kg had a more pronounced trend of tumor volume reduction than those treated with the dose of 5 mg/kg, indicating that HS-2 inhibited tumor growth in a dose-dependent manner. As shown in Figure 7B, the nude mice in all groups were slowly gaining weight, with no significant differences. Therefore, the *in vivo* results show that compound HS-2 can effectively inhibit tumor growth with no significant toxicity.

## Conclusion

Cancer is not sensitive to radiotherapy and chemotherapy, and it has drug resistance and poor prognosis in most patients with advanced cancer (Srinivasan et al., 2015; Lai et al., 2021). HDAC1 and SPOP are closely related to the targeted therapy of cancers, and targeting HDAC1 and SPOP is effective for cancer treatment, which provides new therapeutic ideas and methods for the treatment of RCC. However, to date, there are no reports on dual-targeting inhibitors of HDAC1 and SPOP. In this study, a novel and potent HDAC1/SPOP dual-targeting inhibitor HS-2 was identified using a docking-based virtual screening strategy and molecular dynamics simulation. The interaction analysis showed that HS 1–4 could establish hydrogen bonds with Asp99 and Tyr303 and form ionic bonds with zinc ions to ensure the good docking activity of HS 1–4 with HDAC1. At the same time, the interaction of the compounds with the residues of Tyr87, Met117, Tyr123, Lys129, Asp130, Gly132, Phe133, and Lys134 ensures that it was fully accommodated in the SPOP active pocket. Further *in vitro* inhibition experiments showed that the four compounds exhibited inhibition of HDAC1 in the nanomolar range and SPOP in the micromolar range. Among them, compound HS-2 showed a stronger inhibitory effect on HDAC1 and SPOP than the positive controls. Therefore, HS-2 was used for molecular dynamics simulation. The results of molecular dynamics simulation showed that HS-2 could stably bind to both HDAC1 and SPOP. In the MTT assay, HS-2 showed growth inhibitory activity in the micromolar range against a range of cancer-related cells, which suggested the existence of potentially potent anti-tumor ability of compound HS-2. *In vivo* inhibition experiments showed that compound HS-2 inhibited tumor growth in a dose-dependent manner and had no obvious toxicity. Therefore, these results indicate that HS-2 may be a novel HDAC1 and SPOP dual-targeting inhibitor, which may be of value in the treatment of cancer.

## Data Availability

The original contribution presented in the study are included in the article/Supplementary Material, further inquiries can be directed to the corresponding authors.
