# Supplementary material for: Identification of novel and potent dual-targeting HDAC1/SPOP inhibitors using structure-based virtual screening, molecular dynamics simulation and evaluation of in vitro and in vivo antitumor activity
Source: Front Pharmacol. 2023 Jul 10;14:1208740. doi: 10.3389/fphar.2023.1208740 (PMC10363607; doi:10.3389/fphar.2023.1208740)
Supplement: Supplementary file 1 [file Table3.DOCX]

Supplementary Material

**Identification of novel and potent dual-targeting HDAC1/SPOP inhibitors using structure-based virtual screening, molecular dynamics simulation and evaluation of *in vitro* and *in vivo* antitumor activity**


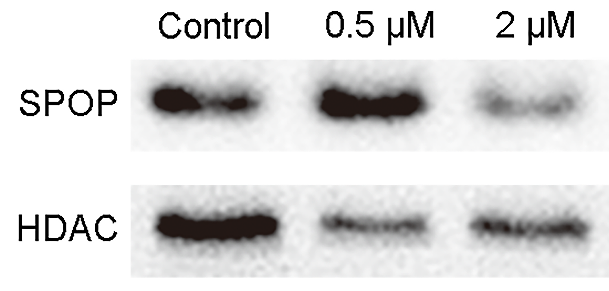


**Figure S1. Effects of HS-2 on SPOP and HDAC in HCT-116 cells.** The SPOP and HDAC levels were measured through western blot.


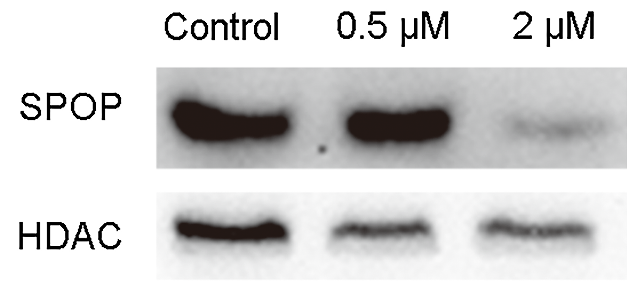


**Figure S2. Effects of HS-2 on SPOP and HDAC i****n A498 cells.** The SPOP and HDAC levels were measured through western blot.
